# Spatiotemporal variability of soil respiration in a seasonal tropical forest

**DOI:** 10.1002/ece3.3267

**Published:** 2017-08-14

**Authors:** Vanessa E. Rubio, Matteo Detto

**Affiliations:** ^1^ Smithsonian Tropical Research Institute Balboa Panama; ^2^ Department of Biological Sciences University of Los Andes Bogota Colombia; ^3^ Department of Ecology and Evolutionary Biology Princeton University Princeton NJ USA

**Keywords:** automated and manual chamber, El Niño/Southern Oscillation, forest structure, spatial and temporal variability

## Abstract

We monitored soil CO_2_ effluxes for over 3 years in a seasonally wet tropical forest in Central Panama using automated and manual measurements from 2013 to 2016. The measurements displayed a high degree of spatial and temporal variability. Temporal variability could be largely explained by surface soil water dynamics over a broad range of temporal scales. Soil moisture was responsible for seasonal cycles, diurnal cycles, intraseasonal variability such as rain‐induced pulses following dry spells, as well as suppression during near saturated conditions, and ultimately, interannual variability. Spatial variability, which remains largely unexplained, revealed an emergent role of forest structure in conjunction with physical drivers such as soil temperature and topography. Mean annual soil CO_2_ effluxes (±*SE*) amounted to 1,613 (±59) gC m^−2^ year^−1^ with an increasing trend in phase with an El Niño/Southern Oscillation (ENSO) cycle which culminated with the strong 2015–2016 event. We attribute this trend to a relatively mild wet season during which soil saturated conditions were less persistent.

## 1 INTRODUCTION

Tropical forests contribute to the global carbon cycle through storing 40% of global terrestrial carbon stocks (Beer et al., 2010; Jobbágy & Jackson, 2000; Pan et al., 2011), 56% of which, is found in aboveground biomass and 32% in soils (Ngo et al., 2013; Pan et al., 2011). For their major role in the global carbon cycle, they will strongly influence future concentrations of atmospheric carbon dioxide (Cox et al., 2013; Sayer, Heard, Grant, Marthews, & Tanner, 2011).

Carbon assimilated by the biosphere is released through autotrophic and heterotrophic respiration (Malhi et al., 1998; Trumbore, 2006) of which respiration from soils is the major component, and second in magnitude only to gross primary productivity (Raich & Schlesinger, 1992). However, the spatial distribution of this carbon source and its sensitivity to global climate change are still uncertain (Pendall et al., 2004), especially in the tropics (Cavaleri, Reed, Smith, & Wood, 2015).

Quantifying the spatial and temporal variability of soil respiration is necessary to estimate ecosystem carbon losses at regional and global scale, and in understanding the mechanisms that control such losses (Phillips et al., 2017), yet, it remains a major challenge (Houghton, 2005; Metcalfe et al., 2007; Schwendenmann & Veldkamp, 2006). Soil is a complex and spatially heterogeneous mixture of minerals and organic pools, including litter, roots, and microorganisms. Each of these components responds differently to environmental variability (Li, Yang, & Fang, 2013) and are uniquely coupled with other biotic processes, such as nutrient recycling (Sayer et al., 2011), generating a broad spectrum of CO_2_ emission rates. It is not surprising that soil respiration rates in tropical forests vary considerably (Table 1), comprising from 41% to 44% (Chambers et al., 2004; Malhi, Doughty, & Galbraith, 2011), up to 60%–80% of the total ecosystem respiration (Goulden, Munger, Fan, Daube, & Wofsy, 1996; Raich & Schlesinger, 1992; Wofsy et al., 1993).

**Table 1:** Mean annual soil CO_2_ efflux (gC m^−2^ year^−1^) in different tropical forests around the world. The annual budgets were computed from the mean soil CO_2_ efflux reported in the study, converted in μmol m^2^ s^−1^ and multiplied by 12 × 10^−6^ × 3,600 × 24 × 365. The length of the study period and the method are also indicated

References	Location	Period	Ecosystem type	Efflux	Method
Wood et al. (2013)	Luquillo, Puerto Rico	6 months	Subtropical wet forest	4,352	Automated chamber IRGA
Valentini et al. (2008)	Northwest Mato Grosso, Brazil	11 months	Upland tropical forest	2,887	Dynamic closed chamber IRGA
Vargas and Allen (2008)	Northeast Yucatan Peninsula, Mexico	16 months	Dense, even‐aged tropical forest	2,876	Solid‐state CO2 sensors
Malhi et al. (1998)	Cuieiras, near Manaus, Brazil	1 year	Lowland terra firme tropical rainforest	2,649	Edisol eddy covariance system IRGA
Sotta (1998)	Manaus, Brazil	2 months	Terra firme wet tropical forest	2,596	Dynamic closed chamber IRGA
Hashimoto et al. (2004)	Chiang‐Mai, Northern Thailand	2 years	Hill evergreen tropical forest	2,560	Portable closed chamber IRGA
Sotta et al. (2004)	Manaus, Brazil	6 months	Lowland terra firme rainforest	2,441	Dynamic open chamber IRGA
Takahashi et al. (2011)	Kanchanaburi province, Western Thailand	3 years	Seasonal tropical forest (lower slope)	2,343	Static closed chamber IRGA
Katayama et al. (2009)	Sarawak, Malaysia	4.6 years	Lowland mixed‐dipterocarp forest	2,214	Dynamic closed chamber IRGA
Ohashi, Kume, Yamane, and Suzuki (2007)	Sarawak, Malaysia	22 months	Primary tropical rainforest	2,013	Dynamic closed chamber IRGA
Adachi, Bekku, Rashidah, Okuda, and Koizumi (2006)	Malaysian Peninsula	2 days	Secondary tropical forest	2,002	Portable closed chamber IRGA
Davidson et al. (2000)	Paragominas, Brazil	15 months	Primary tropical forest	2,000	Dynamic closed chamber IRGA
Adachi et al. (2006)	Malaysian peninsula	2 days	Primary tropical forest	1,985	Portable closed chamber IRGA
Adachi et al. (2005)	Negeri Sembilan, Malaysia	2 days	Primary tropical forest	1,837	Portable system IRGA
Ibañez, (2015)	Nyungwe forest, Rwanda	6 months	Secondary tropical mountain rainforest	1,830	Dynamic closed chamber IRGA
Davidson et al. (2000)	Paragominas, Brazil	1.25 years	Secondary tropical forest	1,800	Dynamic closed chamber IRGA
Adachi, Ishida, Bunyavejchewin, Okuda, and Koizumi (2009)	Western Thailand	2.5 years	Seasonally tropical dry forest	1,724	Portable closed chamber IRGA
Kosugi et al. (2007)	Malaysian peninsula	3 years	Primary lowland mixed dipterocarp forest	1,703	Dynamic closed chamber IRGA
Takahashi et al. (2011)	Kanchanaburi province, Western Thailand	3 years	Seasonal tropical forest (ridge)	1,701	Static closed chamber IRGA
Metcalfe et al. (2007)	Pará State, Brazil	1 year	Lowland terra firme rainforest (Fertile site)	1,699	Dynamic open chamber IRGA
Adachi et al. (2005)	Negeri Sembilan, Malaysia	2 days	Secondary tropical forest	1,691	Portable system IRGA
Zhou et al. (2013)	Southwest of Hainan Island, China	2 years	Primary tropical forest	1,673	Automated closed chamber IRGA
**This study**	**BCI, Panama**	**3 years**	**Lowland tropical forest**	**1,613**	**Dynamic closed chamber IRGA**
Epron et al. (2006)	French Guiana	1 month	Lowland terra firme rain forest	1,612	Dynamic closed chamber IRGA
Ibañez, (2015)	Nyungwe forest, Rwanda	6 months	Primary tropical mountain rainforest	1,570	Dynamic closed chamber IRGA
Jiang et al. (2016)	Southwest of Hainan Island, China	3 years	Primary mountain rainforest	1,567	Automated closed chamber IRGA
Zhou et al. (2013)	Southwest of Hainan Island, China	2 years	Secondary tropical forest	1,510	Automated closed chamber IRGA
Kursar (1989)	BCI, Panama	2 years	Lowland tropical forest	1,506	Chamber‐syringe/Dynamic close chamber IRGA
Sotta et al. (2006)	Pará State, Brazil	2 years	Lowland terra firme rainforest (sandy soil)	1,487	Dynamic closed chamber IRGA
Wu, Goldberg, Mortimer, and Xu (2016)	Yunnan Province, China	1 year	Secondary forest	1,457	Dynamic closed chamber IRGA
Schwendenmann et al. (2003)	La Selva, Costa Rica	2 years	Tropical wet forest (residual soil)	1,425	Dynamic closed chamber IRGA
Giardina et al. (2014)	Mauna Kea Volcano, Hawaii	11 months	Tropical montane wet forest	1,390	Dynamic closed chamber IRGA
Schwendenmann and Veldkamp (2006)	La Selva, Costa Rica	5 years	Tropical wet forest (residual soil)	1,381	Dynamic closed chamber IRGA
Malhi et al. (2011)	–	–	–	1,350	–
Jiang et al. (2016)	Southwest of Hainan Island, China.	3 years	Secondary mountain rainforest	1,300	Automated closed chamber IRGA
Schwendenmann and Veldkamp (2006)	La Selva, Costa Rica	5 years	Tropical wet forest (old alluvium soil)	1,211	Dynamic closed chamber IRGA
Chambers et al. (2004)	Manaus, Brazil	1 year	Old‐growth closed canopy terra firme	1,211	Dynamic closed chamber IRGA
Sotta et al. (2006)	Pará State, Brazil	2 years	Lowland terra firme rainforest (clay soil)	1,166	Dynamic closed chamber IRGA
Schwendenmann et al. (2003)	La Selva, Costa Rica	2 years	Tropical wet forest (old alluvium soil)	1,077	Dynamic closed chamber IRGA
Li et al. (2006)	Luquillo, Puerto Rico	7 months	Secondary wet tropical forest	1,048	Alkali trap method
Sayer et al. (2011)	Gigante, Panama	1 year	Lowland tropical forest	1,000	Dynamic closed chamber IRGA
Fernandes, Bernoux, Cerri, Feigl, and Piccolo (2002)	Rondonia State, Brazil	1 year	Open humid tropical forest	984	Chamber‐syringe method
Kiese and Butterbach‐Bahl (2002)	Queensland, Australia	4 years	Tropical rainforest	835	Automated chamber IRGA
Sha et al. (2005)	Xishuangbanna, China	1 year	Tropical rainforest	831	Static opaque chamber (chromatography)
La Scala, Marques, Pereira, and Corá (2000)	Sao Pablo, Brazil	3 days	Tropical bare soil	792	Dynamic closed chamber IRGA
Mo et al. (2007)	Guangdong Province, Southern China	1 year	Old‐growth monsoon evergreen forest	604	Static chamber (chromatography)

Climatic factors such as precipitation and radiation largely drive temporal variability in soil respiration, influencing soil moisture, temperature, and many biotic processes such as root, soil microbes, and litterfall dynamics. Wet and moist tropical climates are characterized by intense and frequent rainfall events, with or without a seasonal cycle. Soil moisture is probably the most important abiotic factor influencing soil respiration within tropical forests (e.g., Li, Xu, & Zou, 2006; Sotta et al., 2006). Soil CO_2_ efflux can be suppressed in both low and high soil water content (Davidson, Belk, & Boone, 1998; Linn & Doran, 1984). High water content creates a barrier to gas diffusion at the soil‐atmosphere interface, limiting the escape of CO_2_ and supply of oxygen (Liptzin, Silver, & Detto, 2010), thereby reducing both, production and diffusion of CO_2_ (Davidson, Samanta, Caramori, & Savage, 2012; Fang & Moncrieff, 1999). At low soil moisture conditions, decomposition is limited by soluble carbon availability (Davidson et al., 2012; Linn & Doran, 1984).

Several nonlinear relationships have been proposed to link soil respiration rate and soil water content (Cook & Orchard, 2008; Davidson, Verchot, Cattanio, Ackerman, & Carvalho, 2000), indicating optimal conditions for microbial decomposition and root respiration at intermediate moisture conditions. However, these relationships remain empirical, and it is unknown how they vary with soil, climate, and forest type. In addition, these relationships can be altered during rain‐induced pulses, which can be caused by large amounts of water‐soluble carbon leaching from the litter or dead microbes, accumulated during dry periods, known as the “Birch effect.” Although these pulses are often observed in dry ecosystems (Ma, Baldocchi, Hatala, Detto, & Yuste, 2012), are less documented in tropical forests (Cleveland & Townsend, 2006). Because this nonmonotonic and nonlinear response, the effect of changing in rainfall variability is difficult to predict in a particular forest without available observations.

In contrast, soil temperature fluctuations in the tropics are small, especially in areas covered by dense vegetation, where little radiation reaches forest floors. Although temperature is a direct factor affecting root and microbes metabolic rates (Kuzyakov & Gavrichkova, 2010; Lükewille & Wright, 1997) and is responsible for the temporal variation in soil respiration, primarily in temperate (e.g., Hanson, Wullschleger, Bohlman, & Todd, 1993; Vargas, Detto, Baldocchi, & Allen, 2010) and boreal ecosystems (Shibistova et al., 2002), it might play a secondary role in tropical forests (Davidson et al., 2000). For example, results from a rain exclusion plots suggest that the positive effect of temperature on soil CO_2_ efflux is still constrained by soil moisture availability (Wood, Detto, & Silver, 2013).

Relative to temporal variability, spatial variability is less understood, and it can be driven by heterogeneity in below ground physical, chemical, and biological soil properties, landforms, and vegetation cover (Hanson et al., 1993; Xu & Qi, 2001). Spatial variability is also known to be very large at small scales (Epron, Bosc, Bonal, & Freycon, 2006; Kursar, 1989). By shielding intercepting rainfall and determining root distribution, above ground forest structure can create microheterogeneity in the physical and biotic drivers of soil respiration (Raich & Tufekcioglu, 2000). Species composition may also play an important role, as plant species differ in the production and quality of detritus (Raich & Tufekcioglu, 2000), root system, and associations with microorganisms such as fungi and bacterial communities (Barberán et al., 2015). Plant‐soil feedbacks have an important role in the ecosystem nutrient cycling and soil carbon exchange through productivity and carbon input into the soil (Balogh et al., 2011; Bardgett, Freeman, & Ostle, 2008; Sayer et al., 2011). For example, diurnal fluctuations in soil CO_2_ effluxes may also be caused by translocation of photosynthates from leaves to roots (Detto et al., 2012; Kuzyakov & Gavrichkova, 2010).

At larger scales, topographic features (slopes, plateau, and valley) influence hydrological processes and determine heterogeneity in water availability, soil texture, and nutrients (Silver, Scatena, Johnson, Siccama, & Sanchez, 1994; Weintraub et al., 2015). However, the effects on soil respiration are still unclear and literature reports mixed results. Sotta et al. (2006) and Hanson et al. (1993) found no differences between landforms in the Eastern Amazonia and South East US, respectively, while other studies found strong relationship between soil respiration and topographic position, decreasing from hills to bottomlands (Chambers et al., 2004; Epron et al., 2006) or increasing from ridge to lower slopes (Takahashi et al., 2011).

The spatiotemporal variability complicates ground‐based monitoring of soil respiration because of the inherent trade‐off between temporal and spatial sampling resolution. Recent advances in automated systems have greatly improved our ability to monitor temporal variability up to a half hourly resolution, which is comparable to the scale of variation of many climatic and hydrological drivers. However, systems such as dynamic chambers rely on a centralized gas sampling design, which limits their applications in spatially heterogeneous environments. Manual measurements are more adaptable to spatially stratified sampling designs, but sampling frequency is often insufficient to resolve all scales of variation, and there are other logistic problems limiting sampling at night or during, and immediately after rain events. A combination of manual and automated measurements could provide sufficient information to accurately quantify the soil CO_2_ effluxes. Unfortunately, tropical studies that integrate this approach on a sufficiently large temporal horizon are scant (see Table 1).

The objectives of this study were to quantify soil respiration in a lowland seasonally wet tropical forest and analyze their spatial and temporal variability. We used both, manual and automated systems, collecting more than three years of measurements, which include a strong El Niño event. In particular, we investigated the effects of soil moisture, soil temperature, topography, and forest structure. Finally, in order to compute integrated seasonal and annual budgets, we assimilated the measurements in a statistical model at daily scale using Artificial Neural Network.

## 2 METHOD

### 2.1 Site description

The study site is located on Barro Colorado Island (BCI), Panama (9°9′N, 79°50′ W), a 15 km^2^ island in the middle of the Lake Gatun. The forest is tropical moist with a distinct dry season between January and April. Mean annual temperature is 27°C, with minimal seasonal variation; mean annual rainfall is 2642 (±566) mm.

The study was conducted in 6 ha plot at 140 m.a.s.l. on the island plateau. Soil is oxisol containing mainly red light clays, with the majority freely drained, but restricted subsoil permeability giving temporary ponding (Baillie, Elsenbeer, Barthold, Grimm, & Stallard, 2005; Windsor, 1990). The canopy is generally 20–40 m tall estimated to hold 281±20 Mg/ha of aboveground biomass, lianas included (Leigh et al., 2004). In the study plot, The tree density (dbh > 100 mm) at the plot is 287 stem/ha with *Gustavia superba, Alseis blackiana, Trichilia tuberculata, Spondias radlkoferi, Luehea seemannii,* and *Hura crepitans,* as the most common species.

### 2.2 Automated dynamic chamber system

An automated CO_2_ chamber system (LI8100, Licor Bioscience) was installed from June 2013 until January 2015 and from April 2016 to end of August 2016. The system included four automated Dynamic and automatic closed chamber, a multiplexer and a close‐path infrared gas analyzer. Each chamber operated every 20 min and CO_2_ concentration measurements were taken every second for 2 min after the chamber was closed. A 30‐sec prepurge and 45‐s postpurge were introduced to allow flushing the system between each measurement.

The soil collars had an outside diameter of 11.4 cm and were installed 5 cm into the soil 1 month before measurements commenced. The collars were located at the vertices of a 20 × 20 m square centered at the microclimatic tower, where power grid was available.

Data collection was interrupted few times due to power losses (about 33 days total). On May 2014, the pump failed and the sensor was sent for repair and factory recalibration; it was reinstalled on July 2014 until January 2015, when it was designated to another experiment. A new, factory calibrated, identical system was installed in April 2016. The automated system had been operative for 617 days, during which it was visited regularly every 1–2 weeks to check the operative status and clean the chambers from litter or other debris that could prevent perfect closure.

### 2.3 Portable static chamber system

In May 2013, we installed 27 polyvinyl chloride plastic (PVC) rings (25 cm of diameter), inserted at 5 cm of depth and distributed in two different topographic features, plateau and slope, spaced approximately 20 m across the plot (Fig. S1) 1 month before measurements commenced. On August 2014, five additional rings were installed in a recently formed gap, about 800 m north of the plot. The rings were kept free of seedlings during the study period. The lid was made from the bell‐shaped terminal part of the PVC pipe, equipped with gasket to ensure tight sealing. CO_2_ concentrations were measured with a diffuse infrared gas analyzer (Vaisala GMP343) installed on the lid with mounting flange. The probe was calibrated with standard CO_2_ and pure nitrogen gases, approximately every 6 months. Temperature and relative humidity inside the chamber were measured by a Vaisala HUMICAP. Air inside the chamber was maintained well mixed by a small fan operated at 6 V. To avoid pressure differences between the chamber and the atmosphere, the chamber was vented to the atmosphere through a small hole. CO_2_ concentration measurements were recorded by a Vaisala HM70 meter for 5 min at 5 s averaging intervals after closing the chamber. Sampling campaign were conducted at average weekly intervals from June 2013 for a total of 3,847 samples.

### 2.4 Flux calculation and data quality check

Soil CO_2_ effluxes were calculated from regression of CO_2_ concentration within the chamber versus time. For the automated system, effluxes were computed using LI‐8100 File Viewer software (version 3.1.0). The software computed the effluxes using the best model between linear and exponential, based on *R* ^2^. Start time was set at 25 s from the time the chamber was closed according to manufacturer recommendation. Values exceeding reasonable limits (0–16 μmol m^−2^ s^−1^), with *R* ^2^ < 0.97 or RMSE > 0.2 μmol m^−2^ s^−1^ were discarded.

For the manual system, effluxes were computed by linear regression using a graphic user interface developed with MATLAB. Linear trends were selected by visual interpretation, by manually setting the start and end times, which usually implied to discard the first 60 s to ensure that only the linear portion of the curve is used. Values of flux exceeding 16 μmol m^−2^ s^−1^, and poor good of fitness (*R* ^2^ < 0.9 or RMSE > 0.2 μmol m^−2^ s^−1^) were discarded. Other anomalous values were detected by comparing effluxes between consecutive campaigns and discarded if differences between both, previous and following measurements, exceeded 5 μmol m^−2^ s^−1^.

### 2.5 Soil temperature and soil moisture

Continuous soil temperatures were taken in proximity of the automated chambers with four thermistors (model 8150‐203, LI‐COR) of 6 cm length and were operated in conjunction with the chambers. In January 2016, two soil thermistors (Model 107, Campbell Scientific) of 10.4 cm length were installed permanently and recorded by a datalogger (CR1000, Campbell Scientific) at 5‐min interval.

For the manual measurements, soil temperature was taken during four campaigns next to the collars with a platinum thermistor (model HH804U, OMEGA Engineering) inserted at 15 cm of depth.

Soil moisture was monitored by three Time Domain Reflectometers (TDR, Campbell Scientific, CS616) inserted vertically in the soil in proximity of the automated chamber system. Soil moisture measures were taken continuously during the duration of the study. Soil samples were collected between 0 and 15 cm during different soil moisture conditions to calibrate the TDR period against gravimetric measurements. A site specific value of soil bulk density equal 0.75 g/cm^3^ was measured by collecting several soil cores with a metallic cylinder of 10.6 cm diameter and 15 cm height. This value was used to transform mass gravimetric measurements to soil volumetric water content.

Other meteorological variables as solar radiation (CMP11, Keep&Zonen), air temperature and relative humidity (HC2S3, Campbell Scientific), and air pressure (pressure transducer mounted on a LI‐7550, LiCOR) were obtained from the microclimatic tower located in the plot and used as input variables for the Artificial Neural Network (see below).

### 2.6 Forest structure

We recorded the diameter of all the trees at breast height, within 5 m distance from each collar to calculate basal area. We calculated the gap fraction (indirect site fraction) of the canopy above each ring taking hemispherical photographs with a digital camera (Canon EOS 6D, Canon Inc. Japan) provided with a fisheye lens (Sigma 8 mm f/3.5 EX DG Circular Fisheye Lens, Sigma Corporation of America).

### 2.7 Statistical analysis

Correlation analysis was used to determinate the relationship between measurements taken by automated and manual chambers, the spatial relationship between temperature and soil CO_2_ effluxes and the relationship between the magnitude of rain‐induced pulses and soil moisture fluctuations. From the automated time series, ten pulses were selected during the dry seasons and their magnitudes estimated as the percentage increase between the values of the effluxes immediately before the rain event and at the attained maximum.

A quadratic relationship between spatially averaged soil CO_2_ effluxes and soil moisture was fitted using a robust nonlinear least‐squares method implemented in the function *fit* (MATLAB 2014a). The relationships were fitted independently for automated and manual samples.

Coefficient of variation (CV), semivariogram (γ), and autocorrelation functions (acf) were used to quantify the temporal and spatial variability of the effluxes during the entire period and during the dry and wet periods separately (for CV and γ only). The autocorrelation as function of time interval τ was defined as:

(1) $$\text{acf}\left(\tau \right)=\frac{E[\left(f\left(x,t\right)-{〈f〉}_{t}\right)\left(f\left(x,t+\tau \right)-{〈f〉}_{t+\tau }\right)]}{\sqrt{E[{\left(f\left(x,t\right)-{〈f〉}_{t}\right)}^{2}]}\sqrt{E[{\left(f\left(x,t+\tau \right)-{〈f〉}_{t+\tau }\right)}^{2}]}}$$

where E[·] denotes expectation and ${〈f〉}_{t}$ spatial average for all measurements *f* taken at time *t*. The semivariogram as function of spatial lag *r* was defined as:

(2) $$\gamma \left(r\right)=E\left[\frac{1}{2}{\left(\overset{¯}{f}{\left(x\right)}_{T}-\overset{¯}{f}{\left(x+r\right)}_{T}\right)}^{2}\right]$$

where $\overset{¯}{f}{\left(x\right)}_{T}$ denotes temporal average for measurements at location *x* across the period *T*.

Multiway analysis of variance was used to determine the effect on the spatial variability of several factors using the function *anovan* (MATLAB 2014a). The analysis was performed on the residuals between the effluxes and the model with soil moisture fitted in the previous analysis. Significance was determined at *p* < .05.

Diurnal pattern of soil effluxes was computed by averaging continuous measurements as function of time of the day during the 2016 dry season. To compare soil temperature, soil moisture, and soil CO_2_ efflux, the diurnal patterns were normalized between 0 and 1.

### 2.8 Artificial Neural Networks (ANN)

Because manual and automated measurements were acquired at different temporal resolution and irregular frequency, averaging across space and time is problematic. ANN is a statistical technique commonly used to gap fill biological fluxes (gross primary productivity, ecosystem respiration, and evapotranspiration) obtained from eddy covariance measurements (Papale & Valentini, 2003) in order to produce meaningful time integrated budgets. Here, we used ANN with the same scope. All data were assimilated at daily time step into the ANN which uses meteorological inputs (soil moisture, solar radiation, temperature, and pressure) to predict soil CO_2_ effluxes at each location. Once all the series have been put on the same time frame, they were easily averaged.

To train the network, the dataset was randomly divided into a training set (70%), a validation set (15%) and a test set (15%). A two‐layer feed‐forward network with 10 sigmoid hidden neurons and linear output neurons was trained using the Levenberg‐Marquardt algorithm until the mean square error (*MSE*) of the validation set stop improving (Hagan & Menhaj, 1994).Performance, in term of *MSE,* was evaluated using the test set at the end of the training. This procedure was repeated 100 times for each location to produce 100 estimates of daily soil CO_2_ effluxes. Training multiple times generates different results due to different initial conditions and random sampling of the three sets. Ensemble at any locations *i* and day *j* was obtained as weighted average from the 100 ANN predictions using the inverse of *MSE* of the test set as weights according to:

(3) $$f_{ij}=\frac{\sum_{k}f_{ijk}^{ANN}/MSE_{ik}}{\sum_{k}1/MSE_{ik}}$$

The ANN was implemented using the Neural Network Toolbox in MATLAB 2014a.

## 3 RESULTS

**Figure 1:**
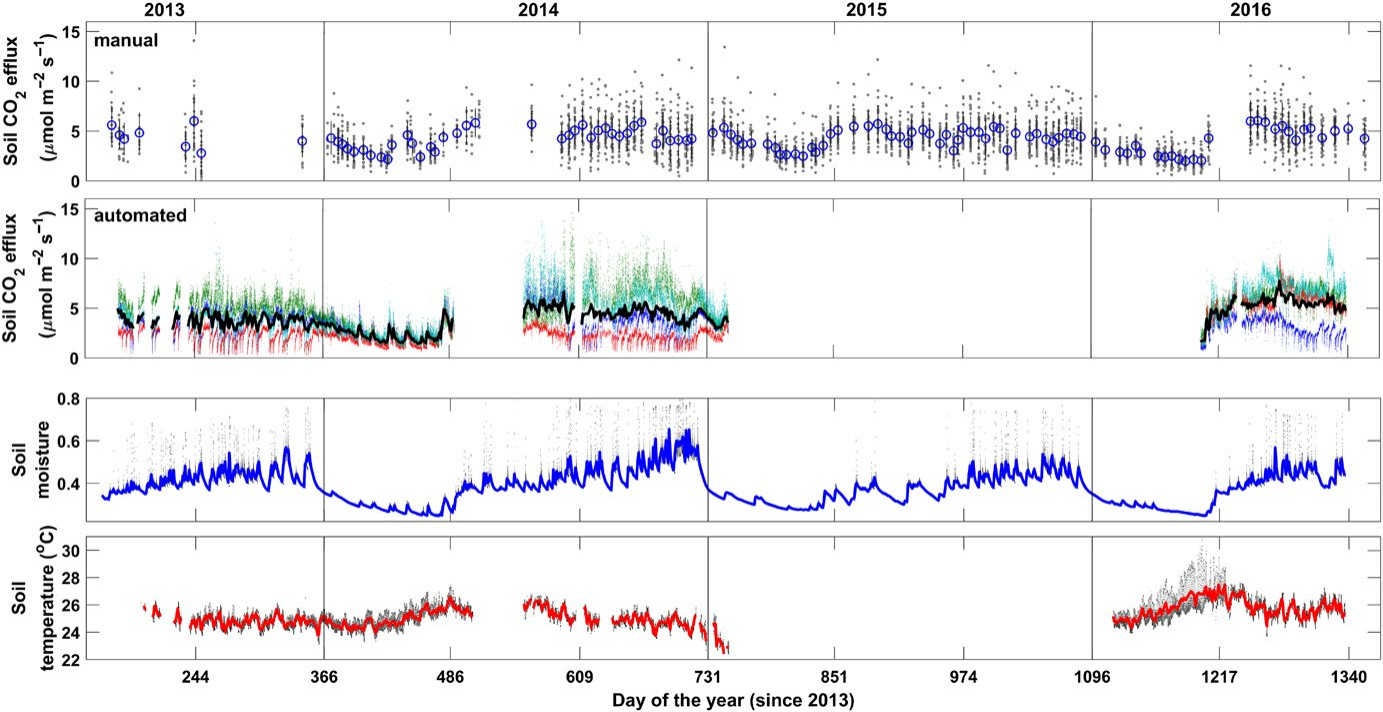
Time series of soil respiration (soil CO_2_ effluxes) for manual (a), four automated chambers (b), soil moisture (c), and temperature (d) collected on Barro Colorado Island between 2013 and 2016. In (a) points represent single measurements, blue circles field campaign mean, and black lines *SE*. In b–d) each point indicated an individual measurement, tick lines are daily means. Gaps were due to instrument malfunctioning, lack of personnel, power losses, and maintenance operations

Time series of soil respiration measurements (Figure 1a,b) showed a clear seasonal pattern in phase with soil moisture (Figure 1c). There was a large spatial variation in the weakly manual measurements and in the four automated measurements. Automated measurements also displayed large temporal variability at finer time scales. Soil CO_2_ effluxes ranged from as low as 0.06 to a maximum of 14.07 μmol CO_2_ m^−2^ s^−1^ and 0.25–14.57 μmol CO_2 _m^−2^ s^−1^, for the manual and automated system, respectively.

In addition to seasonal cycle, surface soil moisture exhibited high‐frequency fluctuations in correspondence to rain events (Figure 1c). Lower values of soil CO_2_ efflux were found when the soil was either dry or completely wet, immediately following heavy rain events in the wet seasons. In contrast, even moderate rain events after long dry spells generated CO_2_ pulses of variable magnitude.

Soil temperature exhibited less variation, both diurnal and seasonal, ranging from 22 to 30 degrees Celsius across the entire record. Highest values were reached during the 2016 dry season in correspondence to a strong El Niño event (Figure 1d).

**Figure 2:**
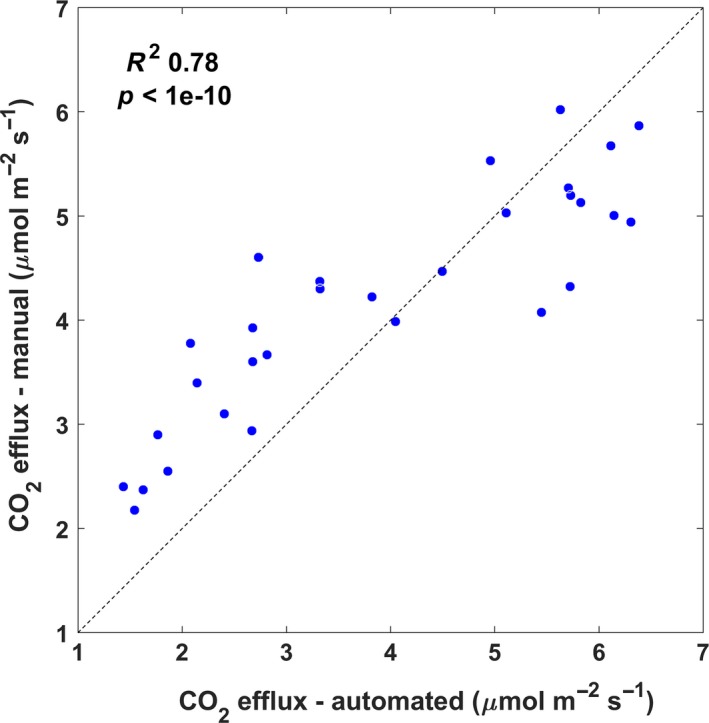
Comparison of soil CO_2_ efflux measurements estimated as average of four automated chambers and average of 32 manual chambers during periods when both systems were operated. 1:1 line shown for reference

Automated and manual measurements were in good agreement when compared on a daily scale. Figure 2 shows a scatterplot representing the average of the 32 manual measurements for each census against the average of the four chambers during the time of the day correspondent to the duration of the field campaign. Although there was a good correlation (*R* ^2^ = 0.78, *p* < 1 × 10^−10^), manual measurements were higher for low soil CO_2_ effluxes and lower for high soil CO_2_ effluxes.

**Figure 3:**
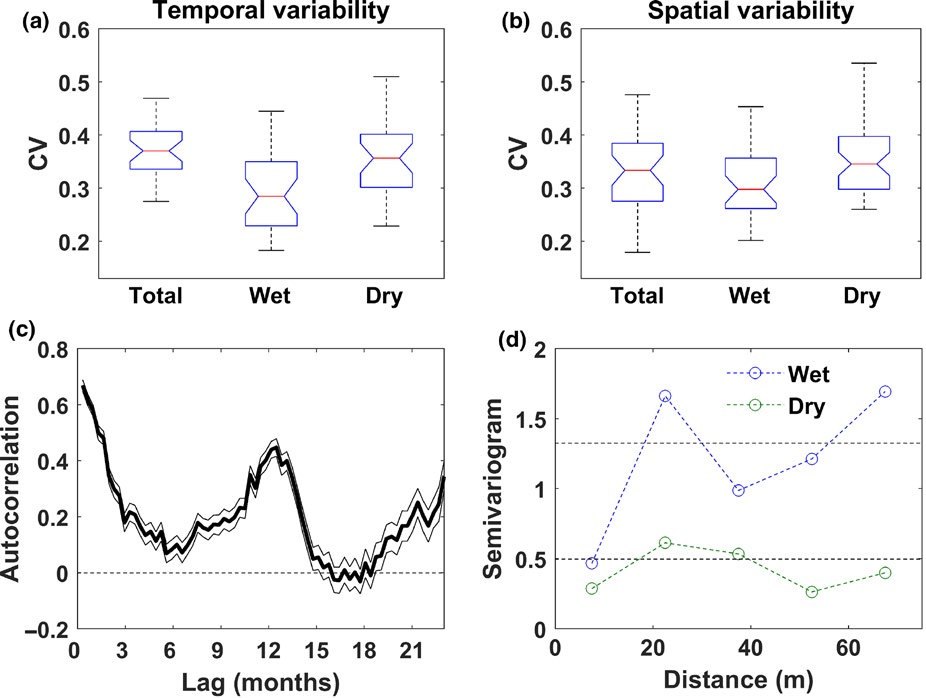
Spatiotemporal variation in the manual measurements shows a large degree of variability in both, temporal and spatial axes, strong temporal autocorrelation and lack of spatial structure. Boxplot of coefficient of temporal variation (CV) of soil CO_2_ effluxes among locations during all periods, wet (swc > 0.35) and dry (swc < 0.35) conditions (a). Boxplot of coefficient of spatial variation of temporally averaged soil CO_2_ effluxes during all period, wet and dry conditions (b). Autocorrelation function (c). Semivariogram during wet and dry conditions (d)

**Figure 4:**
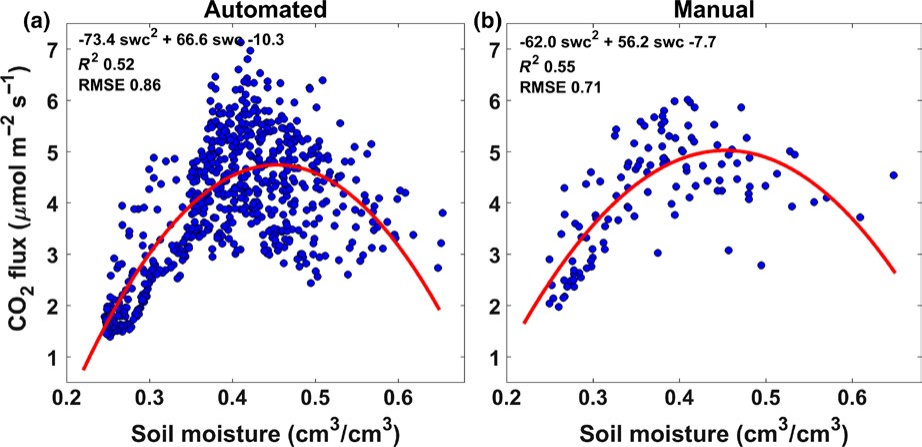
Quadratic relationships between soil moisture and soil CO_2_ effluxes obtained from automated and manual measurements. Blue dots represent daily average flux measurements with daily averaged soil water content between 0 and 15 cm. Fitted equation, *R* ^2^ and root mean square error are also reported

Temporal and spatial variabilities of soil respiration were both high with coefficient of variations (CVs) ranging from 0.27 to 0.46, and 0.17 to 0.47 for temporal and spatial CV, respectively (Figure 3a,b). During the dry season, CVs were higher than during the wet season, indicating strong relative variability during low flux periods. Temporal autocorrelation function showed a long‐term correlation (>15 months) with annual periodicity (Figure 4c). In contrast no spatial structure was detected in the semivariogram (Figure 3d), suggesting that from a 20 m distance, measurements can be considered statistically independent.

The automated and manual measurements showed a consistent quadratic relationship between soil moisture and soil CO_2_ effluxes (Figure 4). For both automated and manual measurements, the peak of soil CO_2_ effluxes was at ~0.45 cm^3^/cm^3^.

**Figure 5:**
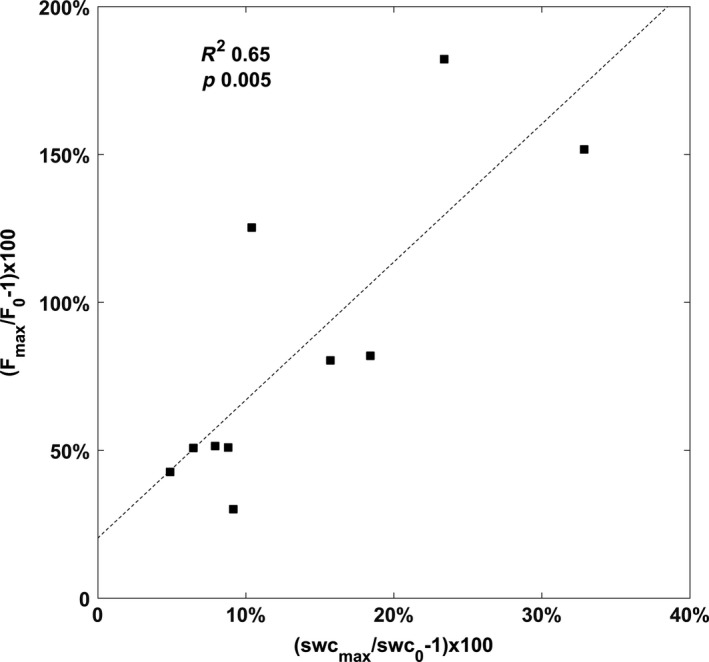
Rain‐induced pulses of soil CO_2_ effluxes (*F*) explained by relative changes in soil moisture (swc). Each point represents a pulse with magnitude expressed as the relative difference of the flux measured just before the rain event (denoted as 0), and the maximum flux of the pulse (denoted as max). Least‐squares line, *R* ^2^ and *p*‐value are shown for reference

Pulses of soil respiration during dry season were strongly correlated with the magnitude of the soil moisture fluctuations, with the soil CO_2_ efflux doubling for a 20% increase in soil moisture (Figure 5).

**Figure 6:**
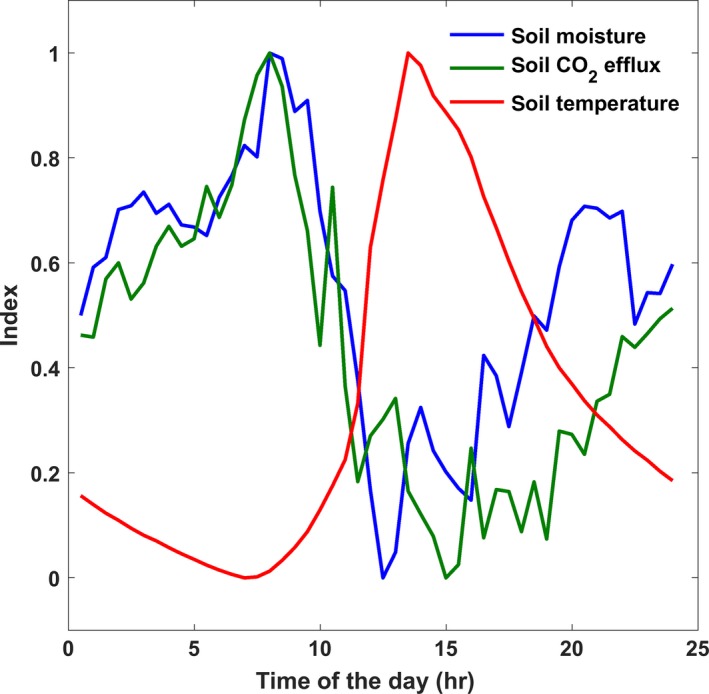
Mean diurnal variation of soil CO_2_ effluxes is out of phase with soil temperature and soil moisture during 2016 dry season. All variables are normalized between 0 and 1 for comparison

Automated measurements allowed us to study diurnal cycles in soil respiration, which were consistently detected during the dry seasons with an average amplitude of ~0.6 μmol m^−2^ s^−1^. This diurnal variation was in phase with soil water content, as illustrated in Figure 6, while temperature was lagging for about 5 hr.

**Figure 7:**
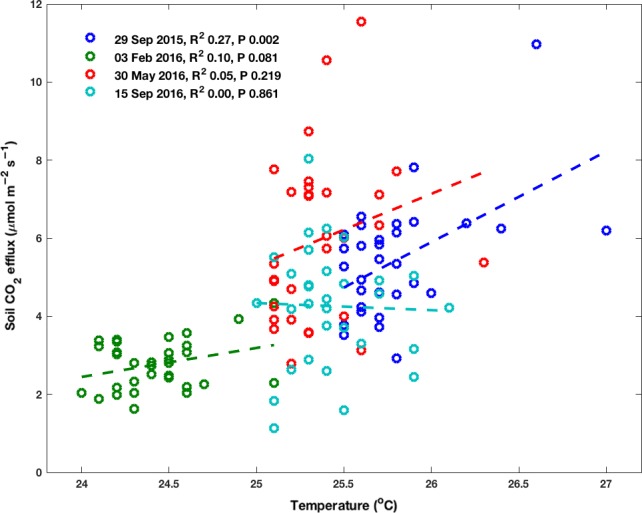
Relationship between manual CO_2_ effluxes and soil temperature measured in the proximity of the collars during four filed campaigns. Date of the census, *R* ^2^ and *p*‐value are indicated in the legend. Linear regressions are indicated by dashed lines for reference

Spatial soil temperature variations were minimal, spanning approximately one degree Celsius in each of the four manual censuses (Figure 7). Not surprisingly, these small spatial temperature differences did not explain much variation in the soil CO_2_ effluxes. The correlation between manual CO_2_ effluxes and soil temperature was significant in one census only (*R* ^2^ = 0.27, *p* = .002, *n* = 32), marginally significant in one census (*R* ^2^ = 0.10, *p* = .081, *n* = 32), and not significant in the other two censuses (*R* ^2^ = 0.05, *p* = .219, *n* = 32; *R* ^2^ = 0, *p* = .861, *n* = 32). When the significant temperature census was included in the multiway ANOVA, the overall effect of temperature was significant, but mostly in conjunction with gap fraction (Table 2).

**Table 2:** Multiway analysis of variance (ANOVA) for testing the effects of multiple factors and their interactions on the mean of the residual soil CO_2_ effluxes (after removing the temporal dependence on soil moisture with a quadratic model)

Variable	Explained variance (%)	Coefficient^a^	*F*‐statistics	*p*‐value
Top	0.06	–	2.23	.1358
BA	4.92	0.528	194	<1 × 10^−10^
GF	1.65	0.927	65	<1 × 10^−10^
Temp	0.58	0.480	22.75	<1 × 10^−5^
Top*BA	5.53	–	217.87	<1 × 10^−10^
Top*GF	0.18	–	7.08	.0078
Top*Temp	0.06	–	2.39	.1222
BA*GF	0.41	−0.192	16.34	<1 × 10^−4^
BA*Temp	0.28	0.157	10.94	<1 × 10^−4^
GF*Temp	4.25	−0.257	167.4	<1 × 10^−10^
Total	12.97			

Top: plateau and slope, BA, log of basal area within 5 m from the collar; GF, gap fraction from hemispherical photos; Temp, soil temperature measured on 29 Sept 2015. All continuous variables are rescaled to unit variance.

a For continuous variables only.

Although within topographic features spatial variation was large (coefficient of variation was 20% and 22% along slope and plateau, respectively), the average soil CO_2_ efflux along the slope was slightly bigger than on the plateau (4.38 ± 0.07 and 4.22 ± 0.05 μmol m^−2^ s^−1^, respectively), and the difference was significant (*p* < 1 × 10^−6^). However, when topography was included in a multivariate analysis with other variables, only the interaction terms were significant, in particular with basal area and gap fraction (Table 2).

The multiway ANOVA (Table 2) also revealed significant effects of basal area, gap fraction, temperature and several interaction terms that explain spatial variation in the manual measurements after removing the temporal dependence on soil moisture. However, the total variance explained by the model was only ~13%.

**Figure 8:**
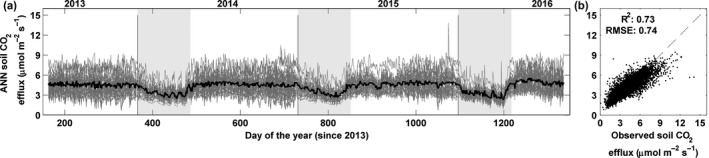
(a) Ensemble ANN predictions at daily time step for each location (gray lines). Tick black line is the average across the 36 locations (32 manual and 4 automated), gray shaded areas indicate dry seasons. (b) Comparison between observed and simulated effluxes. *R* ^2^, root mean square error and 1:1 line are given for reference

Artificial neural network fits the data reasonably well, with an overall RMSE of 0.74 μmol m^−2^ s^−1^ ranging from 0.24 to 1.5 depending on locations (Figure 8). The annual (from August to July) integrated soil CO_2_ effluxes (±*SE*) were 1,591 (±61), 1,602 (±61) and 1,646 (±57) gC m^−2^ year^−1^ for 2013–2014, 2014–2015, and 2015–2016, respectively. Average daily soil CO_2_ effluxes were 3.45 (±0.11) and 4.52 (±0.19) gC m^−2^ day^−1^ during the dry season (January through April) and wet season (May through December), respectively.

## 4 DISCUSSION

Our long‐term observations displayed large spatial and temporal variability, the latter over a broad range of scales, with similar degree of variation among space and time. Despite lower emissions, the dry season exhibited higher relative variability because dry soil responded dynamically to rainfall events as commonly observed in water‐limited ecosystems (Ma et al., 2012), where they can be responsible for more than 10% of the carbon losses over a year (Jarvis et al., 2007). These temporal processes reflected also on the spatial variability, which was relatively higher during dry season most likely because each location responded differently to these rainy events. Automated measurements showed that soil CO_2_ effluxes were greatly reduced immediately following an intense rain event. This effect can last several hours, with a complete recovery of prestorm conditions after approximately 2 days. This means that during the high of the wet season soil respiration is always limited because storm frequency averages one every 2–3 days.

Previous studies highlighted the control of soil moisture on soil respiration (e.g., Davidson et al., 2000; Schwendenmann, Veldkamp, Brenes, O'Brien, & Mackensen, 2003; Sotta et al., 2006; Xu & Qi, 2001). In our study, soil moisture confirmed to be the primary driver of temporal variability in this tropical forest, with temperature playing a secondary role. However, although the effect of soil moisture was well described by a quadratic relationship, large scatter still remains at intermediate moisture conditions, evident from the automated measurements which have higher temporal resolution and a better representation of the rain pulses. The soil CO_2_ effluxes peak, at 0.45 cm^3^/cm^3^, was consistent with other studies that have used a parabolic function to describe the relationship between soil moisture and soil respiration (e.g., Chambers et al., 2004; Sotta et al., 2004). For example, Wood et al. (2013) found higher soil CO_2_ efflux at 0.375 m^3^/m^3^, and Schwendenmann et al. (2003) found that the peak ranged from 0.35 to 0.50 m^3^/m^3^ depending upon the type of soil.

In tropical forests, where temperature variations, both temporal and spatial, are generally small, soil temperature does not significantly influence soil respiration. Actually, the time series displayed an apparent negative correlation (Figure 1). This confounding effect, analogous to what was found by Davidson et al. (1998), is caused by soil moisture to mask the effect of temperature, because the covariation between cold/hot and wet/dry conditions.

In fact, the largest fluctuations in soil temperature were observed during the dry season. In this period, clear sky conditions and canopy opening left by the dry deciduous species, allow larger amounts of solar radiation to reach the forest floor, generating deep diurnal cycles in soil heat. It is in concomitance with these fluctuations that flux measurements showed a detectable diurnal cycle (Figure 7). An in‐depth analysis of the lags between temperature and soil CO_2_ effluxes demonstrated that these cycles were not consistent with a temperature dependent process, because effluxes peaked at 8:00 a.m. while temperature peaked at 13:30 p.m. Considering that gas soil diffusivity is relatively high in dry conditions, it seems unlikely that temperature produced a lagged response of more than 18 hr.

Again, this diurnal pattern is almost in phase with soil moisture, which in the dry season also exhibited detectable diurnal cycles generated by root water uptake. This suggests two possible explanations: (1) Sugars are synthetized during the day and translocated to roots. These sugars are respired by roots or released as exudates in the rhizosphere, and used by soil microorganisms such as mycorrhizae (Kuzyakov & Gavrichkova, 2010). The time of this coupling, which is determined by phloem transport dynamics (Mencuccini & Hölttä, 2010), generates a lagged response between photosynthesis (or transpiration) and soil respiration. (2) Part of the carbon respired by roots can be transported aboveground through the xylem rather than being diffused through the soil (Aubrey & Teskey, 2009), reducing measured soil CO_2_ effluxes during periods of high transpiration. Although this pattern was extremely interesting, the magnitude of the fluctuations was relatively small, and the phenomenon was present only during dry periods, with marginal contribution for annual budgets. For this reason, it was neglected for upscaling manual measurements.

We did not find spatial correlation in our sampling design, which might indicate a lack of large‐scale structure, confirmed by the weak effect of topography. This simplified the analyses because the samples could be considered statistically independent. However, it does not shed light on the spatial scales of variation and the possible mechanisms that generated such variation, suggesting a presence of an unresolved fine scale variability, which might be much smaller that our sparse (20 m × 20 m) sampling design, as previously observed by Kursar (1989).

Although spatial variability was less characterized than temporal variability, our study suggests that forest structure has important direct and indirect influences. For example, the effect of temperature was small but significant, as indicated by the multiway ANOVA, but it was the interactions between temperature and gap fraction that explained most of the variance. This suggests that spatial temperature effects were mediated by canopy structure, which may prove important for upscaling soil CO_2_ effluxes using remote sensing products such as LiDAR. Similarly, higher soil CO_2_ effluxes were observed from slope compared to plateau, a topographic effect generally attributed to hydrological processes related to water transport and drainage (Epron et al., 2006; Ngo et al., 2013; Zhou et al., 2016). However, the multiway ANOVA showed that the topographic effect was mostly significant when interacting with basal area. This might be consistent with the observation that within these forests, mean canopy height, a predictor of above ground biomass, is strongly correlated with hydrological terrain attributes, with taller forests in the proximity of the drainage network (Detto, Muller‐Landau, Mascaro, & Asner, 2013).

Other studies investigated the spatial correlation between the soil CO_2_ efflux and tree proximity, but results were not consistent and the mechanisms unclear. For example, Sotta et al. (2004) found no correlation with basal area. Similarly, Bréchet, Ponton, Alméras, Bonal, and Epron(2011) showed that the soil CO_2_ efflux was poorly explained by forest structure because the contribution of trees to soil functioning depends on both, their quantitative characteristics and qualitative traits. Conversely, Shibistova et al. (2002) found that rates from relatively open areas were about half of those observed below or around trees, a result that was attributed mostly to root density. Vargas and Allen (2008) found that gaps in the canopy could explain fluctuations in soil volumetric water content, causing soils to become wetter or drier faster during or after a rain event, respectively. Strong correlation with forest stand structure at ecosystem scale was also found by Katayama et al. (2009) and Shi, Gao, Cai, and Jin (2016).

The study period was characterized by an ENSO cycle that culminated with a strong El Niño in 2015–2016. During El Niño, this region of the tropics experiences relatively dry and warm conditions. Because the relationship between soil respiration and soil moisture is not monotonic, dry conditions can enhance or reduce annual carbon losses. Our study indicated an increase of annual CO_2_ fluxes during El Niño, mainly driven by a reduction in the frequency of saturated water soil conditions during the wet season. Considering that the frequency and intensity of ENSO are predicted to increase, these results will be useful to understand the impact of climate change on tropical forest carbon cycle.

This study showed how an innovative combination of long‐term automated and manual measurements could help to better quantify soil CO_2_ effluxes in a temporally and spatially variable environment and provide an accurate estimation of landscape soil carbon losses at annual scale. Our soil CO_2_ effluxes fall within the range of the tropical studies presented in Table 1. The annual budget is not statistically different from the mean of the distribution computed from the averaged effluxes reported in these studies (*p*‐value .1608, *t*‐test with 48 dof). However, the length of the study period and the method used for measuring the soil CO_2_ effluxes varies among studies, making meaningful comparison difficult, highlighting the importance of long‐term observations, and standardized method.

Statistical models as the ANN implemented in this study are useful tools to integrate the measurements in space and time, but mechanistic models will be necessary to project soil carbon losses from tropical forests under different climate change scenarios. For this reason, it is essential that future research aims to understand some of the unresolved variability of soil respiration, especially the spatial variability. Important mechanisms that should receive better attention in tropical forests are ecological drivers, as the carbon translocation from above ground to roots and the interactions with soil microorganisms.

## CONFLICT OF INTEREST

None declared.
