# Supplementary figures and images for: Spatiotemporal variability of soil respiration in a seasonal tropical forest
Source: Ecol Evol. 2017 Aug 14;7(17):7104–16. doi: 10.1002/ece3.3267 (PMC5587468; doi:10.1002/ece3.3267)

Appendix

S1. Location of the rings on the plot, for manual and automated system.


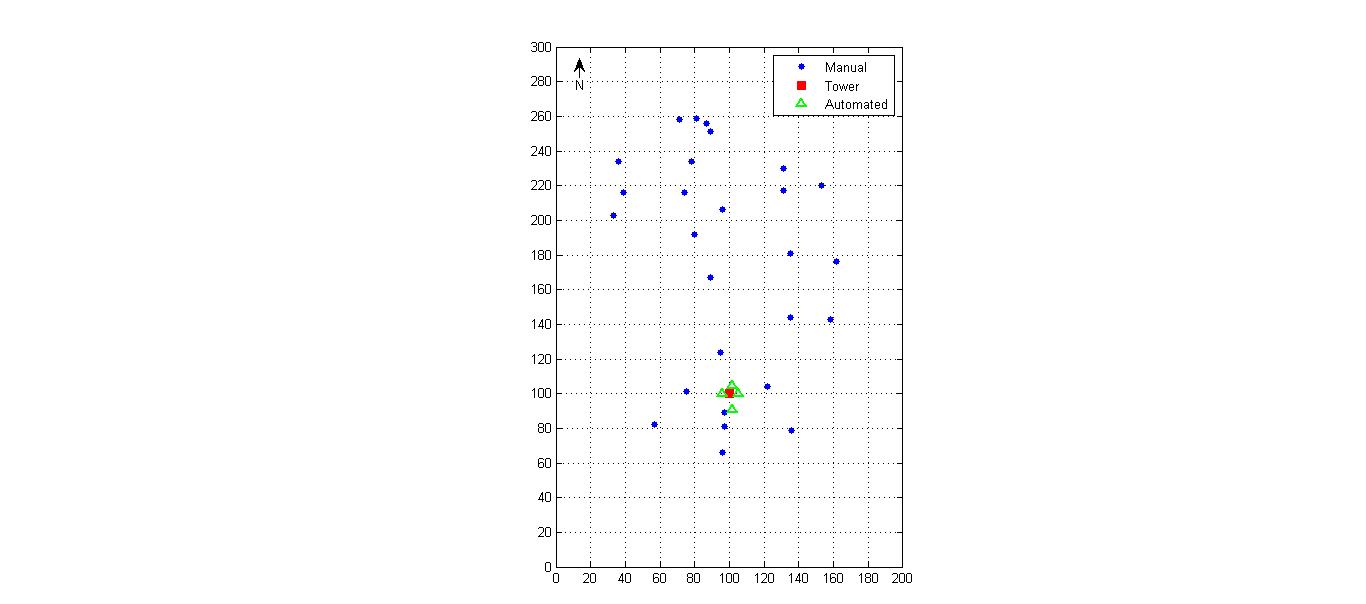

Supplement: Supplementary file 1 [file ECE3-7-7104-s001.docx]
